# Changes of Cerebral and/or Peripheral Adenosine A_1_ Receptor and IGF-I Concentrations under Extended Sleep Duration in Rats

**DOI:** 10.3390/ijms18112439

**Published:** 2017-11-17

**Authors:** Mounir Chennaoui, Pierrick J. Arnal, Rodolphe Dorey, Fabien Sauvet, Sylvain Ciret, Thierry Gallopin, Damien Leger, Catherine Drogou, Danielle Gomez-Merino

**Affiliations:** 1 Institut de Recherche Biomédicale des Armées (IRBA), Département Neurosciences et Contraintes Opérationnelles, 91223 Brétigny sur Orge, France; pierrick.arnal@gmail.com (P.J.A.); dorey.rodolphe@gmail.com (R.D.); fabien.sauvet@gmail.com (F.S.); ciret.sylvain@gmail.com (S.C.); catherine.drogou@gmail.com (C.D.); dangomez51@gmail.com (D.G.-M.); 2 Equipe d’accueil EA7330 VIFASOM, Université Paris Descartes, Hôtel Dieu, (Vigilance Fatigue et Sommeil), 75004 Paris, France; damien.leger@htd.aphp.fr; 3 Laboratoire de Physiologie de l’Exercice, Université de Lyon, 42000 Saint Etienne, France; 4 ESPCI ParisTech, Laboratoire Plasticité du Cerveau, 75005 Paris, France; thierry.gallopin@espci.fr; 5 Centre du Sommeil et de la Vigilance, Hôtel Dieu, APHP, 75004 Paris, France

**Keywords:** sleep extension, A_1_ receptor, IGF-I, hormones, peripheral and brain tissues

## Abstract

Extended sleep improves sustained attention and reduces sleep pressure in humans. Downregulation of adenosine A_1_ receptor (A_1_R) and modulation of the neurotrophic factor insulin growth factor-1 (IGF-I) in brain structures controlling attentional capacities could be involved. In the frontal cortex and hippocampus of rats, we measured adenosine A_1_R and IGF-I protein concentrations after photoperiod-induced sleep extension. Two groups of twelve rats were adapted over 14 days to a habitual (CON) 12:12 light–dark (LD) schedule and an extended (EXT) 16:8 LD schedule. IGF-I content was also measured in plasma, liver, and skeletal muscle. In EXT, compared to CON rats, A_1_R content in the frontal cortex was significantly lower (*p* < 0.05), while IGF-I content was higher (*p* < 0.001), and no significant change was observed in the hippocampus. IGF-I content in plasma and muscle was higher (*p* < 0.001 and *p* < 0.01), while it was lower in liver (*p* < 0.001). The absolute weight and weight gain were higher in EXT rats (*p* < 0.01). These data suggest that 14 days under a 16:8 LD photoperiod respectively down- and upregulated cortical A_1_R and IGF-I levels. This photoperiod induced an anabolic profile with increased weight gain and circulating and muscular IGF-I levels. An extension of sleep duration might favor cerebral and peripheral anabolism, which may help attentional and physical capacities.

## 1. Introduction

There is plenty of evidence indicating that sleep is crucial and is an important component of human life. It has been proven to play an important role in homeostatic restoration, thermoregulation, tissue repair, glymphatic function, immune control, and memory processing [1]. The primordial role of sleep in human physiology and health is exemplified by both observational and experimental research showing that short sleep duration may impair both cognitive function in young and older adults [2,3] and endocrine and metabolism regulation [4], thus potentially representing a challenge to human health. Interestingly, sleep extension, a non-pharmacological countermeasure, limits the consequences of sleep loss on cognitive performance in healthy young adults [5,6,7]. In this population, we recently proved that six nights of sleep extension (~1.2 h per night) improve sustained attention and reduce sleep pressure at baseline and limit degradation and microsleeps during total sleep deprivation and recovery. We initially suggested that upregulation of the neurotrophic factor insulin-like growth factor (IGF-I) and/or downregulation of adenosine A_1_ receptor (A_1_R) may represent the physiological mechanisms of such benefits [6]. After that, we showed that, in healthy men, six nights of sleep extension were without significant effects on mRNA levels of A_1_R and adenosine A_2A_ receptor (A_2A_R) in leukocytes at baseline, during sleep deprivation, and during recovery [8]. 

Adenosine is a metabolic intermediate of the energy-rich molecule adenosine-tri-phosphate (ATP), acting as an endogenous, homeostatic sleep factor, likely mediating the sleepiness that follows prolonged wakefulness [9]. The sleep-inducing effects of extracellular adenosine are mediated mainly through the inhibitory G protein-coupled adenosine A_1_ receptor (A_1_R) and the excitatory G protein-coupled adenosine A_2A_ receptor (A_2A_R) that are ubiquitously distributed throughout brain [9,10]. The A_1_ receptors are widely expressed in the brain in the cortex, thalamus, hippocampus, and basal ganglia [11] and are thought to contribute to sleep induction in a brain-region-dependent manner [9]. Previous studies have shown the involvement of A_1_R and A_2A_R on working memory but with conflicting results due to their different distributions throughout the brain together with divergences in the working memory tasks used [12]. In rats, pharmacologic elevation of adenosine in the basal forebrain has been shown to produce vigilance impairments, and the antagonization of A_1_R reverses them [13]. Acute and chronic sleep deprivation increase extracellular adenosine in the basal forebrain and frontal cortex [14] and upregulates the A_1_R density in several brain regions [15]. 

IGF-I is both a paracrine/autocrine substance produced in various tissues, including the brain, where it plays an important role in brain development and neuron survival and neurocognitive functions in adults [16,17,18]. IGF-1 protein and mRNA concentrations have been quantified in the hippocampus and frontal cortex of adult rats [19]. Circulating IGF-I, derived primarily from the liver, can gain entry into the brain via transport across the blood–brain barrier [20,21]. IGF-I in blood travels as a complex with an insulin-like binding protein 3 (IGFBP-3) and an “acid labile subunit” chaperone that regulates availability to target tissues by regulated release of IGF-I. It has been described that neuronal activity drives IGF-I transport into the central nervous system through the stimulation of matrix metalloproteinase-9 (MMP-9), leading to cleavage of IGFBP-3 (that renders IGF-I free) and interaction with the membrane cargo protein transporter lipoprotein-related receptor 1 (LRP1) [22]. It is mainly the growth hormone (GH) that regulates IGF-I; thereby, many actions of GH are mediated via IGF-I. There are links between the activity of the GH/IGF-I axis and sleep [23]. A chronic deficiency in the entire somatotropic system (using a transgenic mouse with a deficiency of GHRH-GH-IGF-I) was found to be accompanied by sleep loss, particularly non-rapid eye movement sleep (NREMS) [24]. Recently, it was evidenced that IGF-I administration ameliorated the sleep deprivation-induced impairments of cardiac and metabolic indicators in rats [25]. 

In most laboratory rodents, the lengthening of the photoperiod represents an extension of sleep duration [26,27]. Indeed, their sleep–wake distribution shows clear day–night changes under light–dark conditions with high levels of sleep during the light period (rest) and low levels in the dark when the animals are active. We recently showed that 14 days under an extended photoperiod (16 h of light and 8 h of dark, LD 16:8) increased total sleep time and the non-rapid eye movement (NREM) sleep stage per 24 h in rats [28]. A recent study has demonstrated that a long (LD 16:8), relative to short (LD 8:16), photoperiod increased serum IGF-I levels, food intake, lean mass, and growth rate, in photoperiodically sensitive juvenile rats [29]. However, no data seem to exist with respect to the change in brain adenosine receptors or IGF-I under extended photoperiods in rats.

The present study was conducted to characterize the effects of 14 days under extended photoperiods (LD 16:8) on protein content of A_1_R and IGF-I in two rat brain areas, the frontal cortex and hippocampus. IGF-I concentrations in liver and skeletal muscle, and the circulating concentrations of IGF-I, testosterone, corticosterone, prolactin, and insulin, were also determined. We firstly hypothesized that a long photoperiod would respectively down- and upregulate A_1_R and IGF-I content in brain regions controlling attentional capacities and cognitive performance. Secondly, the anabolic effects of the long photoperiod on growth and peripheral hormonal responses were expected.

## 2. Results

### 2.1. Body and Adrenal Gland Weights

The mean (± SD) body weight of EXT rats was significantly higher compared to CON (392 ± 15 g vs. 367 ± 18 g, *p* = 0.002). The mean (±SD) daily weight gain of EXT rats was significantly higher compared to CON (8.21 ± 0.59 g vs. 6.98 ± 1.09 g, *p* = 0.007), and the weights of adrenal glands were not statistically different (46.9 ± 8.0 mg vs. 41.6 ± 9.3 mg).

### 2.2. A_1_R and IGF-I Concentrations (Expressed in pg/mg Protein) in Brain Areas

**Figure 1:**
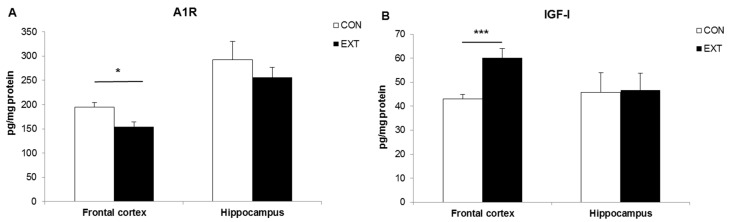
Adenosine A_1_ receptor (A1R) (**A**) and IGF-I (**B**) protein concentrations in the frontal cortex and hippocampus of rats after 14 days of habitual (CON) and extended (EXT) light–dark conditions (respectively 12:12 h and 16:8 h). *n* = 12 per group. Significant difference between CON and EXT: * *p* < 0.05; *** *p* < 0.001.

The mean A_1_R concentration in the frontal cortex of EXT rats was significantly lower compared to CON (*p* < 0.05), while the IGF-I concentration was significantly higher (*p* < 0.001). No statistical difference was observed in the hippocampus (Figure 1A,B).

### 2.3. IGF-I Concentrations (Expressed in pg/mg Protein) in Liver and Skeletal Muscle

The mean IGF-I concentration in the liver of EXT rats was significantly lower compared to CON (*p* < 0.001), while it was higher in the skeletal muscle (*p* < 0.01) (Table 1).

### 2.4. Plasma IGF-I and Hormone Concentrations

The mean IGF-I concentration in the plasma of EXT rats was significantly higher compared to CON (*p* < 0.001), as was the prolactin concentration (*p* < 0.05). No statistical difference was observed for corticosterone, testosterone, and insulin concentrations (Table 2).

### 2.5. Correlation Analysis

The cortical A_1_R levels were significantly and negatively correlated with cortical, plasma, and muscle IGF-I levels. The cortical IGF-I content was positively correlated with plasma and muscle IGF-I content, and negatively with liver IGF-I content. The plasma IGF-I content was positively correlated with muscle IGF-I content, and negatively with liver IGF-I content (Table 3). The plasma prolactin was positively correlated with body weight and body weight gain (*r* = 0.431 and *r* = 0.572, *p* < 0.05, respectively). The weight gain was positively correlated with plasma and muscle IGF-I content (*r* = 0.593 and *r* = 0.408, *p* < 0.05, respectively). 

## 3. Discussion

In this study, our main objective was to investigate the change in A_1_R and IGF-I protein levels in two brain areas of rats after 14 days of extended sleep induced by the photoperiod lengthening (LD 16:8 (EXT) vs. LD 12:12 (HAB)). In addition, changes in the anabolic factor IGF-I were investigated in the main peripheral tissues of action, the skeletal muscle, and the liver, and in the circulation with also those of testosterone, corticosterone, prolactin, and insulin. The results showed that A_1_R and IGF-I levels were, respectively, lower and higher in the frontal cortex of EXT rats, with no significant change in the hippocampus. IGF-I levels were also higher in the plasma and skeletal muscle, and lower in the liver. Finally, circulating prolactin levels were higher in EXT compared to HAB rats, while other hormones were not statistically different. The photoperiod lengthening results in higher body weight gain.

We previously hypothesized that, in healthy humans, cerebral A_1_R and the neurotrophic IGF-I would be, respectively, down- and upregulated over six nights of sleep extension (~1.2 h per night), as sustained attention was improved and sleep pressure was reduced at baseline (before total sleep deprivation) and their degradation was limited during sleep deprivation and during recovery [6]. Anatomical brain structures controlling attentional capacities were suggested to be involved. Indeed, studies in rodents and humans have shown that sleep deprivation affects cortical systems involved in a variety of functions, including executive attention, working memory, and higher cognitive abilities [30]. The A_1_R, which are widely distributed in the brain with particular high concentrations in the cortex and hippocampus, have been sought to be responsible for the sleep-promoting effects of adenosine [9,10,31]. Our hypothesis for a downregulation of A_1_R by extended sleep resulted from a previous study showing that chronic (five days) sleep restriction is associated with upregulation of A_1_R density in cortical and subcortical brain structures in rats [15]. These authors observed a change in A_1_R density within a range of 30% and postulated that this likely has significant functional consequences, notably the long-lasting cognitive impairment related to sleep deprivation. In comparison, they found a significant reduction in A_2A_R density in only one of the three brain areas analyzed, the olfactory tubercle, and no change in the globus pallidus and caudate-putamen, and they did not measure the A_2A_R binding in the hippocampus and frontal cortex because it was low in many areas of the brain. Comparatively, chronic treatment of rats with high doses of nonspecific adenosine receptor antagonist caffeine, the most commonly consumed psychoactive substance worldwide, led to a 17% and 28% increase in A_1_R density [32]. In our study, we suggested that additional sleep over 14 days may have decreased neuronal activity and adenosine tone, resulting in lesser extracellular adenosine levels and consequently lower A_1_R levels in the frontal cortex, which could favor anabolic processes. Indeed, adenosine is considered an indicator of neuronal activity-dependent energy use, by reflecting ATP breakdown [9]. In 2010, Dworak et al. [33] suggested that sleep potentiated anabolic processes because they demonstrated a surge in ATP levels in the initial hours of spontaneous sleep in wake-active brain regions, such as the frontal cortex and to a lesser extent the hippocampus, in positive correlation with EEG NREM delta activity. We found no significant effect of sleep on A_1_R in the hippocampus, which may be due to (i) the number of rats per group, (ii) the characteristics of the extended photoperiod (the daily amount of additional sleep and the number of days), (iii) the measurement in whole hippocampal tissue and not specific areas such as CA1 (Cornu Ammonis 1) or dentate gyrus [15], or (iv) a lower ATP surge as previously observed [33]. Finally, additional work is needed to fully characterize the effects of the lengthening of the photoperiod on A_2A_R protein levels in the frontal cortex and hippocampus because in this study the hypothesis pertains only to A_1_R and IGF-I. Moreover, the sampling of brain tissue was limited.

IGF-1 is a small peptide that is produced not only in the periphery but also in various parts of the central nervous system (CNS) [18]. Moreover, IGF-1 has the ability to cross the blood–brain barrier; thus, the brain is also a target of circulating IGF-1 [20,21]. Circulating IGF-I is derived primarily from the liver and is regulated mainly by GH, which is known to be pulsatile, and pulsatile secretion seems to be important in some GH effects. Conversely, IGF-I reduces serum GH levels (via somatostatin negative feedback in the pituitary), which in turn suppresses GH action in the liver [18,34]. When produced in the brain, IGF-1 exerts combined effects on neural cell signaling and neurotrophic responses, and plays an important role in brain development and neuron survival [16,17,18]. Besides, within the brain, IGF-1 is proteolytically processed by a specific acid protease, generating an N-terminal Gly-Pro-Glu tripeptide (GPE) that plays a role not only in neuroprotection but also in neural repair [35,36]. In adult rats, IGF-1 expression is very high, especially in areas of the brain with large projection neurons, such as the cerebellum, olfactory bulb, hypothalamus, hippocampus, cortex, and retina [18,19]. We found higher levels of IGF-I in the frontal cortex, in the circulation, and in the skeletal muscle in the EXT sleep condition, compared to the CON condition. However, we cannot distinguish if higher cortical levels are consecutive of local production or peripheral entry from blood, even if positive significant correlation exists between them. We have previously shown that circulating levels of IGF-I are higher in the morning after six nights of extended sleep (72 min precisely per night) and remain higher after total sleep deprivation and recovery in healthy men [37]. The chronic increase in GH secretion with extended sleep over the six nights was suggested to have led to the increase in circulating IGF-I. Thereafter, we evidenced that 14 days under extended sleep using photoperiod lengthening led to significant increase in total sleep time and NREM duration per 24 h in rats [28]. In the present study, we thus suggested that higher IGF-I in the frontal cortex may be related to the stimulating effect of longer sleep time and NREM sleep on GH secretion [38], and the consecutive increase of brain IGF-I production and/or circulating IGF-I. The non-significant effect of extended sleep on hippocampal IGF-I levels supported previous results showing that sleep quality and sleep quantity in non-stressed animals showed predictive changes in translational factor activity in cortical but not hippocampal regions [39]. The absence of effect may also be due to IGF-I measurement in whole hippocampal tissue—not specific areas dedicated to learning and short-term memory—and synaptic plasticity such as CA1 or dentate gyrus [40]. In our study, additional sleep time induced higher circulating and muscle IGF-I levels, and higher weight gain was positively correlated with them, and these results shed light on the anabolic properties of sleep [33]. The changes in day length, i.e., the photoperiod, directly modulate the central circadian clock (i.e., the master clock) in the suprachiamastic nucleus (SCN) of the hypothalamus and peripheral clocks in the peripheral tissues, such as the liver, the skeletal muscle, and brain areas other than the SCN [41]. The photoperiod also influences body mass, daily food intake, and energy expenditure, as shown in juvenile Wistar rats [42]. Growing rats exposed to long (LD 16:8) versus short (LD 8:16) photoperiods have a higher rate of weight gain, similar energy intakes, lower growth efficiency, lower daily energy expenditures, and resting metabolic rates and gain more lean body mass [42]. Moreover, GHRH and serum IGF-I levels were found to be photoperiod-dependent in juvenile photoperiodically sensitive Fischer-344 rats, with increased levels of serum IGF-I and hypothalamic GHRH mRNA after long (LD 16:8), relative to short (LD 8:16), photoperiods for a short term (four weeks) [29]. In this study, the long photoperiod is associated with higher body weight, higher food and protein intakes, and higher lean mass. Our results also underlined that body weight gain and IGF-I levels in the blood, frontal cortex and skeletal muscle were higher after long photoperiods, although the content of IGF-I protein stored in the liver was lower. The latter could be explained in part by a downregulation of hepatic mRNA for IGF-I through a negative feedback exerted by the increase of circulating IGF-I on the hypothalamic control of GH secretion [16]. This also allow us to suggest that higher circulating IGF-I levels may originate from local production in all bodily tissues [18]. 

In our study, prolactin levels were significantly higher in rats after EXT sleep compared to HAB, as were IGF-I levels, while no difference was observed for testosterone, corticosterone, and insulin. Additionally, prolactin levels were positively correlated with body weight and weight gain. Prolactin is also a major anabolic hormone [43,44], considered sleep-promoting in laboratory animals [45]. In rats, total sleep deprivation decreased prolactin levels as well as IGF-I and GH, and did not affect corticosterone [46]. The photoperiod has been shown to modify prolactin secretion in animals and in humans [47,48]. Plasma prolactin levels (and IGF-I) were higher after chronic exposure to a long vs. a short photoperiod (LD 16:8 vs. LD 8:16) in adult male goats [47], and after chronic exposure to long (14 h) vs. short (8 h) nights in humans [48]. Our results shed light on the anabolic influence of a short-term lengthening of the photoperiod in young adult male rats due to increases in IGF-I levels in blood, the brain, and skeletal muscle, increases in the anabolic prolactin hormone, and increases in body weight gain. However, we found no effect on the levels of anabolic testosterone in spite of increases in prolactin, which has been shown to be antigonadal but at high clinical or pharmacological levels [44]. The number of rats per group or the characteristics of extended photoperiods may also explain the lack of effect from testosterone levels. Finally, the lack of differences for corticosterone levels and the adrenal gland weight underlined the absence of stress after chronic extension of the photoperiod [49]. However, because we made only single time-point measurements, we cannot exclude a circadian variation in corticosterone levels. 

In conclusion, 14 days under daily light exposure of 16 h compared to 12 h significantly lowered A_1_R protein levels and increased IGF-I levels in the frontal cortex, and generated anabolic peripheral responses in nocturnal Wistar rats. We therefore confirmed the possible involvement of the cortical adenosine A1 receptor and the IGF-I neurotrophic factor as regards the beneficial effects of additional sleep on attentional capacities at baseline and during sleep deprivation in humans [5,6]. Future studies exploring sleep deprivation and cognitive tests with respect to rats are essential. The extension of sleep in rats may be a useful non-pharmacological tool for investigating the physiological mechanisms related to the adenosinergic control of sleep and/or its related effects on cognition or memory. It could also help to understand the relationship between sleep and the GH/IGF-I axis, with respect to cellular and molecular levels, the central nervous system, and the periphery. The coupling of non-pharmacological and pharmacological tools, such as the neuroprotective IGF-I-related tripeptide GPE or analog, might be worth investigating.

## 4. Materials and Methods

### 4.1. Animals

Male Wistar rats (Centre d’élevage R Janvier, Le Genest-Saint-Isle, France) (aged 6 weeks and weighed 221 ± 3 g) were brought to the laboratory two weeks before the onset of experiments to acclimatize to the environment. Rats were individually kept in sound-attenuated ventilated chambers (ambient temperature (22 ± 2 °C) and relative hygrometry (50 ± 15%), with ad libitum access to food and water, and under 12:12 h light/dark (LD) cycles (lights on at 8 a.m., 300 Lux). After the two weeks, rats were randomly distributed into two experimental groups and maintained over 14 days under two LD schedules: one group remained under LD 12:12 and the other under LD 16:8 (*n* = 12 per group). They were daily weighted. The experiments were performed in accordance with the European Directive 2010/63/EU and approved by the institutional ethics committee for animal experimentation (CEA Sauvet_2014, 4 September 2014).

### 4.2. Blood and Tissue Processing

The day after the 14th LD schedule, between 10:00 and 11:30 a.m., the rats were decapitated, trunk blood was collected and centrifuged at 3000× *g* for 10 min to separate plasma, and aliquoted plasma was frozen at −80 °C until use. The frontal cortex, hippocampus, liver, and skeletal muscle (gastrocnemius) were dissected on ice and frozen in liquid nitrogen, and then stored at −80 °C until use. The adrenal glands were also removed and weighed because adrenal hypertrophy in rodents potentially marked negative physiological adaptation to the stress condition of chronic and intensive exercise [50]. 

Brain areas were homogenized in 2 mL Precellys CK14 tubes containing 5 V of Hepes buffer (Hepes 25 mM, pH 7.4; Chaps 0.1%; MgCl_2_ 5 mM and protease inhibitor cocktail Sigma P2714, Saint-Quentin-Fallavier, Isère, France) using Precellys Evolution (Bertin Technologies, Montigny-le-Bretonneux, France). Two cycles of 20 s at 5500 rpm were performed with the Precellys Homogenizer, and tubes were placed on ice within the two cycles. Liver and muscle were homogenized in 10 V of Hepes buffer (Hepes 25 mM, pH 7.4; Chaps 0.1%; MgCl_2_ 5 mM and protease inhibitor cocktail Sigma P8340) in 2 mL CK14 tubes and 7 mL CK28 tubes twice for 20 s at 5500 rpm and 8000 rpm, respectively. The homogenates were centrifuged for 20 min at 12,000× *g* (+4 °C). Total protein concentrations were measured in each supernatant using a UV microvolume spectrophometer (Biodrop, Cambridge, UK) for the brain sample and the Bradford method for the liver and muscle. Supernatants were aliquoted and stored at −80 °C until further assays.

### 4.3. Assays of Hormones in Blood

Corticosterone, IGF-1, testosterone, prolactin, and insulin concentrations were determined in plasma by enzyme-linked immunosorbent assays (ELISA) using commercial kits (Arbor Assays, MG100 R&D Systems, Spi-Bio and RayBiotech, Le Perray en Yvelines, Yvelines, France). The limits of sensitivity were as follows: 0.017 ng/mL for corticosterone, 3.5 pg/mL for IGF-1, 0.03 ng/mL for testosterone, 0.2 ng/mL for prolactin, and 5 µUI/mL for insulin. The intra- and inter-assay coefficients of variation were as follows: 4.8% and 9.9% for corticosterone, 4.1% and 4.3% for IGF-1, 2.9% and 6.8% for testosterone, 10% and 13.4% for prolactin, and 10% and 12% for insulin.

### 4.4. Assays of IGF-1 and A_1_ Receptor (A_1_R) in Tissues

IGF-1 and A_1_R were determined in supernatant by ELISA kits (MG100 R&D Systems, Lille, Nord-Pas-de Calais, France and LSBio, Nanterre, Haut-de-Seine, France). For IGF-1 determination, the suitable dilutions of brain, liver, and gastrocnemius supernatants were 1:2, 1:25, and 1:5 respectively. A_1_R concentrations were determined in the frontal cortex and the hippocampus supernatant. The limit of sensitivity was 0.064 ng/mL, and the intra- and inter-assays coefficients of variation were 10% and 12%.

### 4.5. Statistical Analysis

Data are reported as means (±SEM or ±SD), and the statistical analysis was performed through a non-parametric Mann–Whitney test. Spearman rank correlations were run between biochemical and morphological parameters of all rats. Statistica version 10 (Statistica StatSoft, Inc., Tulsa, OK, USA) was used for all data analysis. Cutoff for statistical significance was set to *p* = 0.05. 

## Figures and Tables

**Table 1:** IGF-I protein content in the liver and skeletal muscle (gastrocnemius) of rats after 14 days of habitual (CON) and extended (EXT) light–dark conditions (respectively 12:12 h and 16:8 h).

Tissue Contents	IGF-I	
	CON	EXT
Liver (pg/mg protein)	674 ± 47	377 ± 10 ***
Skeletal muscle (pg/mg protein)	84 ± 7	102 ± 5 **

*n* = 12 per group. Significant difference between CON and EXT, ** *p* < 0.01, *** *p* < 0.001.

**Table 2:** Hormonal circulating concentrations in rats after 14 days of habitual (CON) and extended (EXT) light–dark conditions (respectively 12:12 h and 16:8 h).

Hormones	CON	EXT
Corticosterone (ng/mL)	6.26 ± 1.26	6.32 ± 0.72
Prolactin (ng/mL)	22.60 ± 4.66	41.18 ± 6.36 *
Testosterone (ng/mL)	6.79 ± 1.01	7.52 ± 0.97
Insulin (µU/mL)	34.3 ± 6.1	49.9 ± 6.4
IGF-I (pg/mL)	1109 ± 38	1354 ± 39 ***

*n* = 12 per group. Significant difference between CON and EXT: * *p* < 0.05, *** *p* < 0.001.

**Table 3:** Spearman correlation values (rank coefficient (*r*) and *p*) between brain and peripheral biological variables.

Variables	A1R Cortex	IGF-I Cortex	IGF-I Liver	IGF-I Muscle	IGF-I Plasma	Prolactin Plasma
A1R Cortex	-	−0.635 *	0.344	−0.430 *	−0.461 *	−0.323
IGF-I Cortex	−0.635 *	-	−0.415 *	0.347	0.522 *	0.240
IGF Liver	0.344	−0.415 *	-	0.058	−0.406 *	−0.427 *
IGF-I Muscle	−0.430 *	0.347	0.058	-	0.546 *	0.070
IGF-I Plasma	−0.461 *	0.522 *	−0.406 *	0.546 *	-	0.263
Prolactin Plasma	−0.323	0.240	−0.427 *	0.070	0.263	-

*n* = 24. Significant at * *p* < 0.05.
